# Drivers of Microbial Risk for Direct Potable Reuse and de Facto Reuse Treatment Schemes: The Impacts of Source Water Quality and Blending

**DOI:** 10.3390/ijerph14060635

**Published:** 2017-06-13

**Authors:** Rabia M. Chaudhry, Kerry A. Hamilton, Charles N. Haas, Kara L. Nelson

**Affiliations:** 1 Civil and Environmental Engineering, University of California, Berkeley, CA 94720, USA; rabia@alum.mit.edu (R.M.C.); karanelson@berkeley.edu (K.L.N.); 2 Engineering Research Center for Re-Inventing the Nation’s Urban Water Infrastructure (ReNUWIt), Berkeley, CA 94720-1710, USA; 3 Drexel University Department of Civil, Architectural, and Environmental Engineering, 3141 Chestnut Street, Philadelphia, PA 19104, USA; haas@drexel.edu

**Keywords:** reclaimed water, direct potable reuse, quantitative microbial risk assessment (QMRA), norovirus, *Salmonella*, *Cryptosporidium*, blending

## Abstract

Although reclaimed water for potable applications has many potential benefits, it poses concerns for chemical and microbial risks to consumers. We present a quantitative microbial risk assessment (QMRA) Monte Carlo framework to compare a de facto water reuse scenario (treated wastewater-impacted surface water) with four hypothetical Direct Potable Reuse (DPR) scenarios for Norovirus, *Cryptosporidium*, and *Salmonella*. Consumer microbial risks of surface source water quality (impacted by 0–100% treated wastewater effluent) were assessed. Additionally, we assessed risks for different blending ratios (0–100% surface water blended into advanced-treated DPR water) when source surface water consisted of 50% wastewater effluent. De facto reuse risks exceeded the yearly 10^−4^ infections risk benchmark while all modeled DPR risks were significantly lower. Contamination with 1% or more wastewater effluent in the source water, and blending 1% or more wastewater-impacted surface water into the advanced-treated DPR water drove the risk closer to the 10^−4^ benchmark. We demonstrate that de facto reuse by itself, or as an input into DPR, drives microbial risks more so than the advanced-treated DPR water. When applied using location-specific inputs, this framework can contribute to project design and public awareness campaigns to build legitimacy for DPR.

## 1. Introduction

Many arid urban centers in developed and developing countries are struggling to meet their long-term water needs. In arid regions such as the southwestern US, the situation is exacerbated by drought and changing weather patterns, making surface water supplies less reliable. As an example, a three-year drought left California’s largest reservoirs at below half capacity in 2014 because they are supplied by Northern Sierra watersheds [1]. In the warm and semi-arid regions of the southwestern US, the volume of wastewater produced in urban municipalities is about 50% to 60% of the total water supplied [2]. If treated and reused, this reclaimed water can become a sizable component of the water portfolio of an urban center and can increase resiliency to climate change impacts.

**Figure 1:**
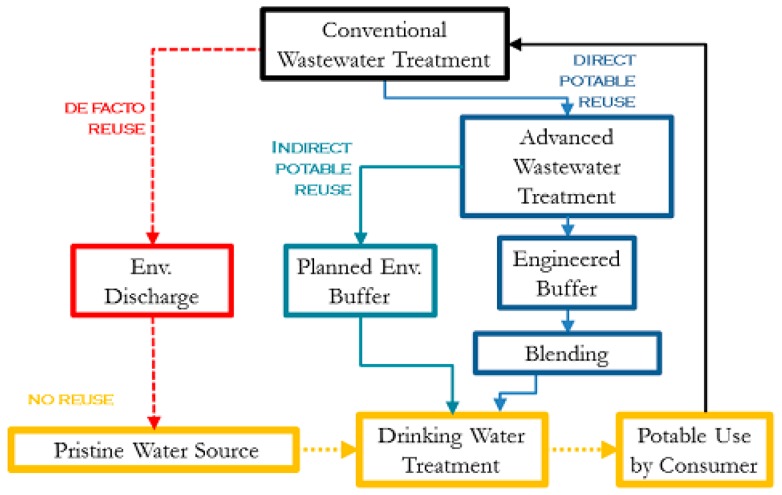
Potable reuse paradigms showing unintended de facto reuse and Direct Potable Reuse with blending prior to conventional drinking water treatment. This paper explores the impact of surface water quality (de facto reuse) and blending on the overall microbial risk to the consumer.

Water reuse in an urban setting can occur in many forms. Both direct and indirect potable reuse paradigms provide an alternative water source for stressed urban centers without the need for costly dual-distribution systems. Figure 1 demonstrates the various paradigms of potable reuse. De facto reuse occurs when the drinking water supply intake for a community is located downstream of a wastewater treatment plant (WWTP) discharge of another community [3]. This form of reuse is unintentional, is often not officially recognized, and is ubiquitous in many parts of the US [3]. In dry summer months, some rivers can become primarily wastewater effluent discharged from upstream facilities [4]. Planned reuse can be for either potable uses, such augmenting the community drinking water supply, or for non-potable uses, such as landscape irrigation [5]. 

Planned potable reuse can further be classified into indirect (IPR) and direct reuse (DPR). Both IPR and DPR involve augmenting drinking water supplies with reclaimed water, but the presence of an environmental buffer such as aquifer recharge distinguishes indirect from direct potable reuse. Use of such a buffer can dispel public distaste for water originating from sewage, and also provides storage to synchronize demand with supply [4]. Several municipalities in the US and around the world successfully practice IPR, including Orange County, California; Fairfax County, Virginia; Gwinnett County, Georgia; and Gerringong, New South Wales, Australia [6]. DPR does not require the presence of an environmental buffer, and existing water supplies can be augmented directly, either upstream or downstream of drinking water treatment, and with the option to include an engineered buffer. The main motivation for DPR is avoiding the infrastructure and cost associated with recharging groundwater or transporting the water to a reservoir.

Although the use of reclaimed water for potable applications has many potential benefits, its use poses concerns for both chemical and microbial risks to consumers. Unlike chemical contaminants of concern in reclaimed water, monitoring of microbial pathogens is a challenge because their detection limits are typically much higher than the concentrations deemed to be safe. Concerns about pathogen removal are the critical driver in water reuse system redundancy and reliability. The implementation of direct potable reuse systems is in the early stages in the US, although DPR has been practiced in Windhoek, Namibia for several decades [7,8]. Success of potable reuse, particularly DPR, depends on reliable pathogen removal, and decision-makers need quantitative tools to compare different treatment scenarios based on consumer health risk, cost, and other utility-specific concerns. 

Quantitative microbial risk assessment (QMRA) can be used for the purposes of estimating the human health risk associated with exposure to waterborne pathogens using a process of hazard identification, exposure assessment, dose response, and risk characterization [9]. Although currently no national regulations for direct potable reuse exist in the United States, microbial quality targets for IPR in California and DPR in Texas use a goal of 1 infection per 10,000 people per year as a risk benchmark to inform the 12–10–10 pathogen log-reduction values for enteric viruses, *Cryptospordium*, and *Giardia*, respectively [10,11,12,13]. Previous work has modelled the microbiological health risks of various water reuse treatment trains [12,14] as well as de facto reuse [15]. However, the microbiological impact of blending highly-treated reclaimed water produced from DPR with conventionally treated effluent impacted surface water (de facto reuse) has not been explored. Investigating this gap is important for exploring the significance of source water quality and blending ratios, and developing a logical rationale for public health-informed blending targets. This is especially relevant as blending is currently being practiced immediately upstream of drinking water treatment facilities in the United States, or in the case of Windhoek, Namibia, downstream of a drinking water treatment within the drinking water distribution system [6,16]. Additionally, no direct comparison has been made between de facto “status quo” and DPR risks; contextualizing DPR risks within this framework may prove valuable for informing water reclamation projects in the future as water scarcity continues to increase.

The research objectives for this study were to: (1) compare the microbial risks for four different DPR configurations alongside a de-facto reuse scenario; (2) show the impact of individual DPR treatment processes to demonstrate the ones that have the greatest potential for health risk mitigation; (3) quantify the effect of wastewater effluent-impacted source water quality on health risks during the blending process; and (4) quantify the impact of blending treated reclaimed water (DPR) water with conventionally treated de facto reuse water for understanding the impact of blending ratio upstream from the conventional drinking water treatment process.

## 2. Materials and Methods 

### 2.1. Hazard Identification

Norovirus (virus), *Salmonella* spp. (bacteria) and *Cryptosporidium* spp. (protozoan parasite) were chosen as representative reference pathogens from each class of water-borne pathogens. Disease caused by these pathogens constitutes a significant portion of waterborne illnesses in the United States [17]. Additionally, occurrence, removal, and dose response information was available in the literature for these pathogens.

### 2.2. Treatment Train Scenarios

Planned reuse can be implemented via countless technological variations. The choice of the treatment scheme depends on many factors, such as capital costs, operation and maintenance costs, expertise required for operation, health risks to the public, disposal of concentrates, energy consumption, and other considerations [4,18]. Each municipality considering reuse must evaluate a plethora of factors before making a decision about the most suitable treatment scheme design for their specific situation. 

Five treatment configurations representing a spectrum of water reuse scenarios were chosen for modeling purposes based on a range of options currently in use, or slated for increased future use, as well as the authors’ professional judgement (Table 1) [6]. Scenario (1) represents de facto reuse. In this scenario, effluent-impacted source water is treated using conventional drinking water treatment comprised of chemical coagulation, flocculation, sedimentation, and filtration through granular media, followed by chlorine disinfection and delivery to the consumer. For the de facto reuse scenario (and scenarios involving blending with de facto reuse water), various source water qualities were considered by accounting for the dilution of treated secondary wastewater in the receiving water body. Pathogen fate and transport was not accounted for as a specific wastewater discharge and/or water body was not considered. The dilution ratio (ranging from 0 to 100%) was considered to act as a bulk parameter for source water quality. Scenarios (2), (3), and (5) all have conventional wastewater treatment as a first step consisting of primary sedimentation and biological treatment with activated sludge. Scenario (2) treatment is followed with microfiltration (MF), reverse osmosis (RO), ultraviolet (UV) advanced oxidation (AOP) (UVAOP/UV and hydrogen peroxide quenching), and chlorination. Scenario (3) conventional wastewater treatment is followed by ozonation (O_3_), biological activated carbon (BAC), MF, RO, UVAOP, and chlorination. Scenario (4) consists of primary sedimentation, membrane bioreactor (MBR), RO, UVAOP, and chlorination, while scenario (5) consists of O_3_, BAC, MF, nanofiltration (NF), UVAOP, BAC, and chlorine. 

The modeled scenarios represent the typical operational variability in treatment at a plant using the specific set of conditions described in the input data. These scenarios also do not account for catastrophic failure of a unit process, such as membrane tearing, which would shut the system down and rely on treatment redundancy to protect consumers. 

The DPR scenarios (2)–(5) model risks only for the advanced-treatment processes mentioned and do not include blending or additional conventional water treatment that would occur in a functioning plant (Table 1). Effects of source water quality and blending are considered separately and described in Section 2.5. Blending highly treated DPR effluent with raw source water for drinking may occur in-line, in a holding tank or in a reservoir within the plant. For modeling purposes, it is assumed that untreated source water is blended with highly treated DPR effluent just upstream of the conventional water treatment processes, resulting in instantaneous and perfect mixing.

### 2.3. Pathogen Concentrations in Raw Wastewater

Recent reviews [19,20] have addressed the occurrence of norovirus in wastewater. Bootstrapped estimates of norovirus (genogroups pooled) in raw sewage from North America gave a mean of 10^3.95^ ± 10^1.10^ gene copies per L [19]. Several authors report concentrations of *Salmonella* in raw wastewater [21,22,23] and a lognormal distribution was fit to pool datasets from the two studies where individual sample data were provided [22,23]. Mean (and standard deviation values reported for *Cryptosporidium* in wastewater influent at a full-scale water reclamation facility in St. Petersburg, Florida were used [24]. Pathogen concentrations are summarized in Table 2.

### 2.4. Pathogen Removals through Unit Process Treatment

For each unit treatment process, the most representative removal and inactivation data for the target pathogens were collected through an extensive literature review (Table 3). Studies that measured actual pathogen concentrations and log removal values (LRV) in full-scale facilities were preferred and used when possible. However, pathogen surrogates and pilot-scale or bench-scale studies were used when no other information was available. In addition, studies that provided multiple data points for log removal or pathogen concentration were preferred so the treatment variability for the unit process could be modeled.

#### 2.4.1. Conventional Drinking Water Treatment Processes

Conventional drinking water treatment processes were modelled including coagulation/sedimentation, granular media filtration, and chlorine disinfection. For *Cryptosporidium*, Harrington et al. [25] conducted challenge experiments with *Cryptosporidium* and *Salmonella* using water from a Wisconsin municipal pilot plant with dual-media filtration columns. Log removal values of 2 ± 0.5 and 1.77 ± 0.25 were calculated from a combination of coagulation, flocculation, sedimentation and filtration of *Cryptosporidium* and *Salmonella*, respectively. The maximum (4.3 LRV) of the modeled distribution is consistent with estimates from a previous review of up to LRV of 4 from conventional drinking water treatment plants [47]. The modelled maximum LRV for *Salmonella* of 2.7 is comparable with estimates up to the LRV of 3 derived from *E. coli* data [48,49]. Norovirus removal through conventional drinking water treatment was modeled using a study of bench experiments using a coagulation-rapid sand filtration process with recombinant norovirus virus-like particles in river water [26]. The coagulant dose was 40 µM-Al of alum [26]. The estimated mean LRV (2.1) is consistent with LRVs reported for bacteriophage MS2 from conventional drinking water treatment (between 1.5 and 2) [48]. 

Chlorination removals during conventional drinking water treatment were calculated based on wastewater removal values measured by several studies. Samples taken at a full-scale plant in Florida were used to calculate *Cryptosporidium* removals of 0.41 ± 0.4 logs. Log removals from Francy et al. [27] during full-scale wastewater treatment for *E. coli* (mean equivalent to median 2.57 LRV, maximum LRV assumed equivalent to 95th percentile at 3.15) and somatic coliphage (median 1.68 LRV, maximum 2.08 LRV) were assumed to be conservative inactivation estimates for drinking water treatment for *Salmonella* and norovirus, respectively. F-specific coliphage data were reported by Francy et al. [27], but were not used as some values were below the detection limit for calculating the log removal. It was necessary to rely on coliphage data because limited information was available for norovirus inactivation with free chlorine at pilot or full-scale facilities. Additionally, Francy et al. [27] did not specify whether free or combined chlorine was used.

#### 2.4.2. Conventional Wastewater Treatment Processes

Conventional wastewater treatment was considered to consist of a primary sedimentation process, biological activated sludge, and granular media filtration. Rose et al. [24] report 1.14 log reduction of *Cryptosporidium* from biological treatment at a full-scale water reclamation facility in Florida. Similarly, Ottoson et al. [28] reported a mean ± standard deviation of 1.58 ± 1.30 *Cryptosporidium* log removal from a pilot plant in Stockholm, Sweden and Cheng et al. [50] reported removals ranging from 0.67 to 1.54 LRV. Due to the similarity in these estimates, the distribution that accounted for variability [28] was used. For norovirus, data from a full-scale plant were used with an estimate of 2.1 ± 0.78 LRV [29]. A distribution of *E. coli* removals from Ottoson et al. [28] was used to represent *Salmonella* removal, consistent with point estimates for removals in various systems [51,52].

#### 2.4.3. Advanced DPR Treatment Processes

For the DPR advanced treatment processes, microfiltration (MF), reverse osmosis (RO), UV, ozone, biological activated carbon filtration (BAC or BAF), membrane bioreactors (MBR), and nanofiltration (NF) were the processes considered. Microfiltration log removal values of 4.6 ± 0.96 were reported by Hong et al. [31] for *Cryptosporidium* from a pilot-plant with 0.2 µm nominal pore size polypropylene filter in Tampa, Florida. This is consistent with a 4–7 log removal range reported by Reardon et al. [53] for protozoa. In a bench-scale microfiltration experiment using recombinant norovirus particles with a PVDF pore size of 0.1 µm, Matsushita et al. [32] observed LRV of 0.6 ± 0.1. For *Salmonella*, Hong et al. [31] also observed an LRV of 5.96 ± 1.47 for *E. coli*. This is assumed to be representative of *Salmonella* in the absence of other data and is consistent with the range reported for bacteria of up to 9 LRVs [53].

#### 2.4.4. Reverse Osmosis Treatment Process

Reverse osmosis removal values for *Cryptosporidium* were reported for pilot-scale RO plants challenge tested with non-viable *Cryptosporidium* oocysts. A mean and 95th percentile value were derived from log-removal values under compromised membrane condition (4.5) or uncompromised (5.7) membrane conditions, respectively [33]. For norovirus, Governal and Gerba [34] conducted challenge tests of MS2 in a pilot-scale system with RO. The mean influent and effluent concentrations for MS2 were 2.2 × 10^5^ ± 1.3 × 10^5^ and 1.2 × 10^1^ ± 0.6 × 10^1^ PFU/ml, respectively, and previously described error propagation techniques [54] were used to determine a removal distribution (µ = 4.3, σ = 0.34). MS2 was considered to be representative of norovirus removal. Data from a Gram negative bacterium (*Klebsiella terrigena*) from a point-of-use RO-iodine resin water treatment system was used to model *Salmonella* removal [35]. 

#### 2.4.5. Ultraviolet and Ozone Disinfection Processes

UV-inactivation was measured in bench-scale experiments with *Cryptosporidium* spiked into samples of filtered water from a water treatment plant for a range of UV doses (1.8–112 mJ/cm^2^) and times; samples were tested using a neonatal mouse model for infectivity [36]. Log removal values were averaged over the doses provided yielding a mean and standard deviation of 2.2 ± 1.17. For norovirus, a bench-scale study of MS2 inactivation in tap water with and without H_2_O_2_ using 127 mJ/cm^2^ UV dose was used to determine a mean LRV of 4.96 ± 0.85 for a UV-only trial at flow conditions of 2 gpm. To obtain a 4-log reduction for virus, 186 mJ/ cm^2^ dose is required for virus, whereas for *Cryptosporidium*, a 22 mJ/cm^2^ dose is sufficient, and suggesting that the log removal used for *Cryptosporidium* is likely very conservative [37]. Francy et al. [27] reported median and maximum removal for *E. coli* of 3.82 and 4.38, which were considered representative of *Salmonella*. Francy et al. [27] did not provide a UV dose, but it is noted that UV dosing at a DPR plant is likely to be an order of magnitude higher than in a conventional wastewater treatment plant, to achieve target removals of trace organics by AOPs. Data from Sherchan et al. [37] was used for norovirus as opposed to Francy et al. [27] because these values were below the detection limit for assessing log removal for norovirus. The maximum LRV was used as the 95th percentile in this assessment to derive a normal distribution for both parameters. The estimated LRVs from these references are within a 0.5 to 4.0 log inactivation range for various UV doses provided by USEPA [55] (original reference Table 1.4). While it is recognized that the LRV is sensitive to the UV dose, the guidance manual allows only point estimates to be determined at each UV dose, and does not provide information on process variability that will occur in full-scale facilities. 

Exposure to 5 mg-min/L ozone achieved 1-log inactivation in bench-scale experiments by Korich et al. [56]. Limited full-scale information was available, however, and treatment due to ozone from a Montreal treatment plant was >1.2 logs for the ozone treatment stage, therefore a point estimate of 1.2 logs was used. Specific information about the ozone dose was not provided. Removal for male-specific (MS2) coliphages was estimated by Tanner et al. [39] as 5.4 logs at 0.22 mg-min/L ozone [12]. Tanner et al. [39] also estimated 4.15 log reductions in *E. coli* occur at 0.25 mg-min/L ozone [12].

#### 2.4.6. Membrane Bioreactor (MBR) Wastewater Treatment Process

MBR removal values were obtained for *Cryptosporidium* from Marti et al. [43] from a pilot MBR with a pore size of 0.4 µm. Sulphite-reducing *Clostridium* spores were used as a surrogate; log removal values were plotted against transmembrane pressure (TMP) without any evidence of correlation. The data point for TMP at 123 mbars was chosen as it represented the greatest variability of 4.3 ± 0.6 log removals. Chaudhry et al. [44] conducted a study of removal of norovirus GII in a full-scale membrane bioreactor with a nominal pore size of 0.04 µm, finding a LRVs of 5.10 ± 0.41. In a study of MBR removal from five full-scale wastewater plants, Francy et al. [27] reported median and maximum removal for *Enterococcus* of 6.26 and 7.49 LRVs, respectively. *E. coli* data were not used as effluent concentrations were below the detection limit for calculating the log removal. The maximum was used as the 95th percentile in this assessment to derive a normal distribution for both parameters. *Enterococci* were considered representative of *Salmonella*.

#### 2.4.7. Unit Process LRV Literature Gaps

Several areas were particularly challenging for assigning LRVs. In particular, BAC had limited information available. As a result, assumptions from a previous risk assessment were maintained for the removals from all the index pathogens [12]. For NF, in the absence of other data, ultrafiltration values were used. Beauchamp et al. [45] conducted a study of log removal values based on integrity pressure-decay tests (detecting holes of 3 µm or more) in a full-scale ultrafiltration plant. These values were used to define a *Cryptosporidium* log removal distribution of 5.52 ± 0.51. Gómez et al. [46] conducted measurements in pilot-scale plants using secondary treated (after activated sludge) wastewater for sand filtration and ultrafiltration using *E. coli*. A LRV of 4.8 ± 0.6 was estimated and considered representative of *Salmonella*. Using recombinant norovirus particles in a bench-scale filtration apparatus (UF regenerated cellulose with a molecular weight cutoff of 1 kDA), Matsushita et al. [32] conducted triplicate experiments with dead-end filtration of river water, observing LRV of 4 ± 0.1. 

### 2.5. Risk Model

A QMRA was conducted to capture typical operational variation using a previously derived framework [57]. Equations for de-facto reuse water and DPR water without considering blending into additional drinking water processes are given by Equations (1) and (2), respectively, where reference pathogen *ref* = norovirus, *Cryptosporidium*, or *Salmonella* arriving at the consumer (*C_consumer,ref_*):

(1) $$C_{consumer,ref}=10^{-L_{DW,ref}}\left[F_{dilution}C_{\mathrm{ww},\mathrm{raw}}10^{-L_{WW,ref}}\right]$$

(2) $$C_{consumer,ref}=\;C_{ww,raw}10^{-L_{DPR,\;t,ref}}$$

For scenarios where de-facto water and DPR water are blended and further downstream drinking water treatment is considered after the DPR trains, the risks were modelled using Equation (3):

(3) $$\begin{array}{ll} C_{consumer,ref}= & 10^{-L_{DW,ref}}\{\left[F_{dilution}F_{blend}C_{ww,raw}10^{-L_{WW,ref}}\right]\\ & +\left[\left(1-F_{blend}\right)C_{ww,raw}10^{-L_{DPR,\;t,ref}}\right]\} \end{array}$$

where *C_ww,raw_* = concentration of pathogen *ref* in raw wastewater influent (pathogens per L), *F_dilution_* = Dilution factor for conventionally treated wastewater effluent in pristine surface water, *F_blend_* = Portion of surface water blended with DPR effluent water prior to conventional drinking water treatment *L_DW_* = total log removals of pathogen *ref* due to conventional drinking water treatment, *L_ww_*= total log removals for pathogen ref due to conventional wastewater treatment, and *L_DPR_* = total log removals for pathogen *ref* due to a given treatment train. The log removals of pathogen *ref* due to treatment processes are given by Equations (4) through (6):

(4) $$L_{DW}=\;\prod_{1}^{n}P_{i,ref}$$

where *P_i_* = log reductions for reference pathogen *ref* for conventional drinking water treatment train removal process *i*;

(5) $$L_{WW}=\;\prod_{1}^{n}P_{i,ref}$$

where *P_i_* = log reductions for reference pathogen *ref* for conventional wastewater treatment train removal process *i*;

(6) $$L_{DPR}=\;\prod_{1}^{n}P_{i,ref}$$

where *P_i_* = log reductions for reference pathogen *ref* for a given DPR train removal process *i.*

The following cases for Equation (3) are considered:

*a*. When *F_blend_* = 0, only DPR trains are considered and no blending with de facto reuse source water is considered, but DPR effluent undergoes additional conventional drinking water treatment;
*b*. *F_blend_* = 1, only de facto reuse is considered;
*c*. For 0 < *F_blend_* < 1, DPR water is blended with de facto reuse source water prior to conventional drinking water treatment.

For cases *b* and *c*, 0 < *F_dilution_* < 1 where 0 is pristine, pathogen-free source-water, and 1 is source water comprised of 100% conventionally-treated wastewater effluent. In a 2008 USEPA model of the top 25 most impacted drinking water treatment plants in the nation, under low streamflow conditions, wastewater effluent constituted between 7 and 100% of the total source water volume [58]. For this reason, in the absence of the use of a specific waterbody, *F_dilutio_*_n_ was simulated over the entire potential range of qualities from 0 to 100% effluent-impacted source water. It is recognized that utilities must comply with outgoing effluent requirements; this is a hypothetical case to explore a range of possible scenarios under various source water conditions and understand the primary drivers of microbial risk when DPR is considered. A daily dose for each reference pathogen was computed using Equation (7):

(7) $$D_{ref}=C_{consumer,ref}V_{ing}$$

where *D_ref_* = daily dose of reference pathogen ref and *V_ing_* = volume of drinking water ingested per day. The daily probability of infection for each pathogen *ref* was computed using dose response models provided in Equation (8) (*Cryptosporidium*), (9) (*Salmonella*), and (10) (norovirus):

(8) $$P_{inf,daily,Cr}=1-e^{-rD_{Cr}}$$

where *r* is the exponential dose response parameter [9];

(9) $$P_{inf,daily,Sa}=1-{\left(1+\frac{D_{Sa}}{\beta }\right)}^{-\alpha }$$

where α and β are parameters of the Beta-Poisson dose response model [9];

(10) $$P_{inf,daily,No}=P\left(1-e^{\frac{-D_{No}}{µ}}\right)$$

where Equation (10) describes the fractional Poisson model [59] and *P* and *µ* are parameters of the model describing the perfectly susceptible fraction of secretor status positive (Se+) individuals and mean aggregate size, respectively. Both aggregated and disaggregated forms of the norovirus dose response model are considered in order to provide a range of predicted outcomes as the selection of norovirus dose response model has been shown to yield meaningful differences in risk estimates at low norovirus doses [60]. Exposure and dose response parameters are summarized in Table 4.

Total daily infection risks for each reference pathogen were calculated according to Equation (11). A similar approach has been used to pool risks from multiple pathogens by previous QMRA studies [12,61,62].

(11) $$P_{inf,\;daily,total}=1-\overset{n_{ref}}{\underset{1}{\prod }}\left(1-P_{inf,\;daily,ref}\right)$$

### 2.6. Risk Characterization

Annual risk was calculated as per Equation (12), where *n* is the yearly frequency of the activity:

(12) $$P_{inf,\;annual,ref}=1-\overset{n}{\underset{1}{\prod }}\left(1-P_{inf,daily,ref}\right)$$

A sensitivity analysis was conducted to identify variables contributing to uncertainty using 100,000 Monte Carlo iterations. All computations were performed in R (www.rproject.org) and using the mc2d package [64]. Random sampling of daily risks with replacement was conducted as per the preferred method for annualizing probability of infection using 100,000 iterations [65].

The Spearman rank correlation coefficient was used to identify the most important predictive factors of annual infection or clinical severity infection risk, were 0 is no influence and −1 or +1 when the output is wholly dependent on that input. The model inputs were ranked based on their correlation coefficient with the output variable, annual risk.

## 3. Results

### 3.1. Comparison of Microbial Risks for DPR Configurations and de Facto Reuse

**Figure 2:**
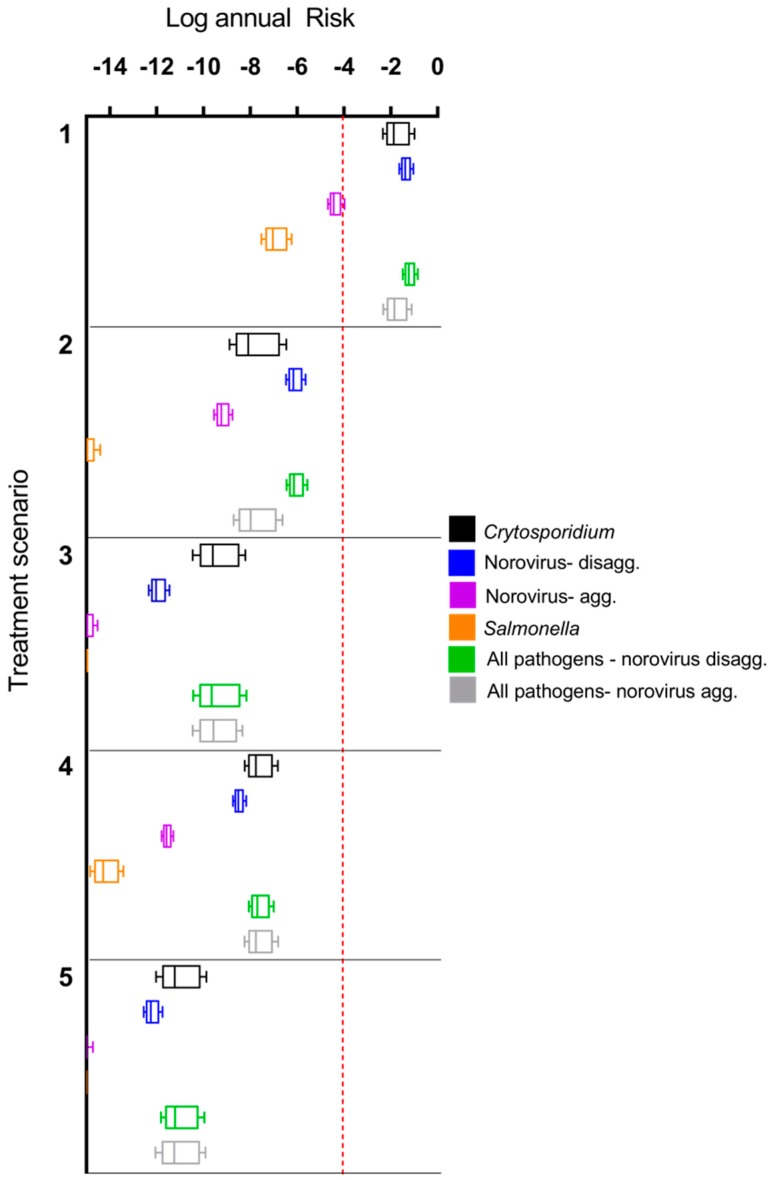
Comparison of annual risks for treatment scenario (1) (de facto reuse) and direct potable reuse (DPR) scenarios (2) through (5), without additional downstream conventional drinking water treatment for the DPR trains. The de facto reuse scenario assumes that 10% of surface water is comprised of secondary treated wastewater effluent. Boxes show 25th, 50th, and 75th percentiles while whiskers extend to the 5th and 95th percentiles. Note that *Salmonella* annual infection risks for Train 5 are <10^−14^.

A comparison of annual risks for treatment scenarios (1) through (5) is shown in Figure 2. The de-facto reuse scenario (1) shown is for a low-level contamination scenario (source water comprised of 10% treated secondary wastewater effluent; on the low end of the 7–100% range given by Rice et al. [58]). With a small degree of wastewater impact to the source water, risk from de-facto scenarios were up to 11 orders of magnitude higher than DPR treatment train effluents. Furthermore, the risk shown for DPR treatment trains in Figure 2 do not include further log reductions due to downstream drinking water treatment [6]. The only scenarios above a 10^−4^ annual risk benchmark were the de-facto reuse scenarios for *Cryptosporidium*, norovirus (using a disaggregated dose response model), and a combination of all three pathogens (*Cryptosporidium*, norovirus, and *Salmonella*). Use of an aggregated vs. a disaggregated dose response model for norovirus had a difference of approximately 3 orders of magnitude in annual risk, with aggregated risks being lower. The rank order of median annual risks computed for the various treatment trains scenarios was: de-facto reuse > scenario (2) > scenario (4) > scenario (3) > scenario (5). Total pathogen risks were dominated by *Cryptosporidium* and norovirus estimates; however, the rank order of these risks varied depending upon the treatment train and dose response model used for norovirus. For scenarios (3)–(5), the risk from norovirus (disaggregated) was lower than *Cryptosporidium*, but the opposite was true for scenarios (1)–(2). Due to lack information on norovirus aggregation behavior during full-scale treatment processes, the difference in aggregation state was considered for the dose response models but not for the LRV parameters. 

### 3.2. Impact of Treatment Stages on Annual Risk

**Figure 3:**
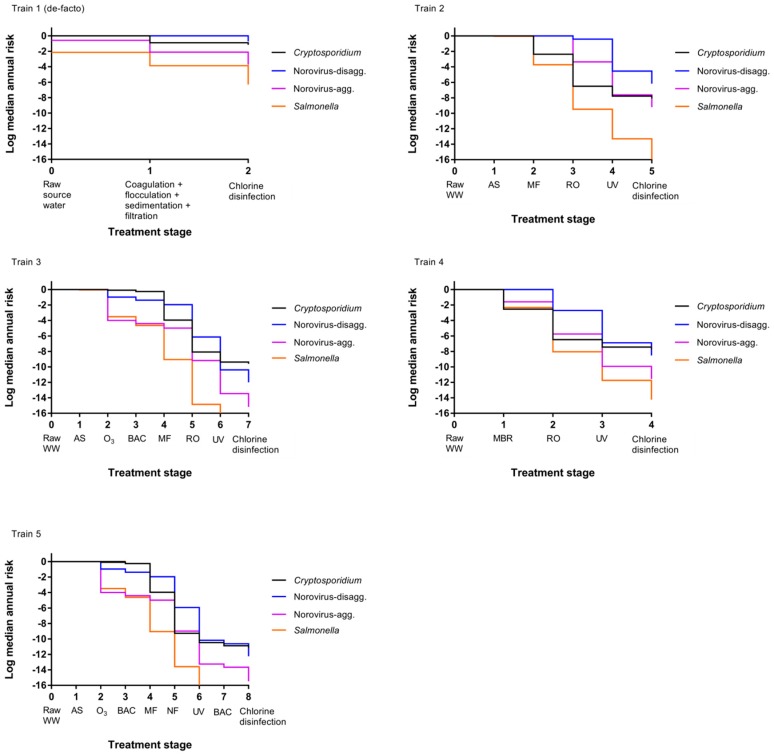
Change in median annual risk at each treatment stage. Train 1 (de-facto) assumes source water comprised of 50% secondary treated wastewater effluent. See Table 1 for a description of direct potable reuse (DPR) Trains 2 through 5.

A comparison of annual risks of each treatment train as they progress through the treatments processes is shown in Figure 3. Train 1 (de-facto reuse) has lower starting risks for *Salmonella* and norovirus-aggregated compared to the other treatment trains because the starting matrix is secondary treated wastewater effluent-impacted surface water rather than raw wastewater, and the concentrations of pathogens are therefore lower. Additionally, no reduction in median annual infection risk for the activated sludge process is visible for Train 2 through 5 and minimal reductions are observed for norovirus-disaggregated in some other early treatment stages. In the early treatment stages, concentrations of pathogens can still be high after a given treatment process, and the risk may still therefore be close to 1 even if there has been a pathogen reduction, resulting in only small or nonexistent differences in Figure 3. 

This comparatively small reduction in earlier treatment trains is especially apparent for the norovirus-disaggregated estimates (for example, Figure 3 train 2). This is due to the fact that the Fractional Poisson disaggregated dose response model will predict higher risks at low doses compared to other available norovirus dose response models; risk curves for scenarios modeled by van Abel et al. [60] using the disaggregated model show a flatter curve when the dose is below 100 gene copies compared to scenarios modeled using the aggregated model (see original reference Figure 1). It is worth noting that several studies have documented virus aggregation in natural waters [41,44,66].

**Figure 4:**
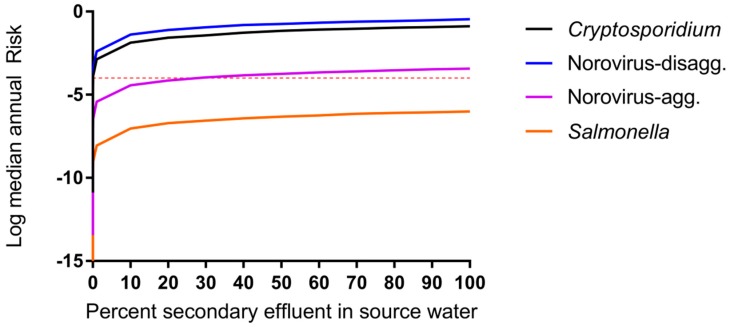
Impact of surface water quality on DPR risk modeled as percent of source (surface) water comprised of secondary treated wastewater effluent (Treatment train scenario (1), de facto reuse, case b, *F_blend_* = 1, 0 *< F_dilution_* < 1). Median annual risks shown for each pathogen. Microbial risk is driven by surface water quality even when supplies are impacted by 1% secondary treated wastewater effluent.

The predicted risks will therefore be high with the disaggregated model until the dose reaches a lower level, at which point the risk decreases more rapidly, and it is easier to see the risk difference from one treatment stage to the next. Membrane-based treatment processes were generally the most effective at reducing pathogen risk in all scenarios. RO had the most significant impact on risk reduction for trains (2) through (4) while MF and NF had the most significant impact on removal in train (5). However, it is noted that the benefit of RO removal is less influential for disaggregated norovirus risk estimates due to the caveats related to the norovirus-disaggregated dose response model described above [60]. A sensitivity analysis is shown in Table 5 for the DPR scenarios and supports that RO is an influential predictor of protozoan and virus risks (Spearman rank correlation coefficient ranging from −0.28 to –0.44 for *Cryptosporidium*, −0.07 to –0.32 for norovirus) but is less influential for *Salmonella* removal (−0.04 to –0.15). In all cases, the removal processes were more influential than the starting concentration of pathogens in wastewater and the rate of drinking water consumption (in L per day consumed) was not an important predictor. Removal by UV, conventional wastewater (activated sludge, settling, and filtration) processes, MBR, and MF were also influential treatment steps for their respective treatment trains. For conventional surface water, the degree of wastewater impact will vary depending on local conditions. To explore the effect of surface water quality on risks due to de facto reuse, annual risks were simulated over the full range from zero to 100% wastewater effluent, without considering die-off or additional pathogen sources to the surface water (Figure 4). By definition, the lowest risk was observed for 0% effluent in surface water or for pristine, pathogen-free source water. The greatest increase in annual risks occurred between 0% and 10% effluent-impacted source water and little increase in risk was observed beyond 20% effluent-impacted source water.

### 3.3. Impact of Blending Advanced-Treated DPR Water and Wastewater-Impacted Surface Water

**Figure 5:**
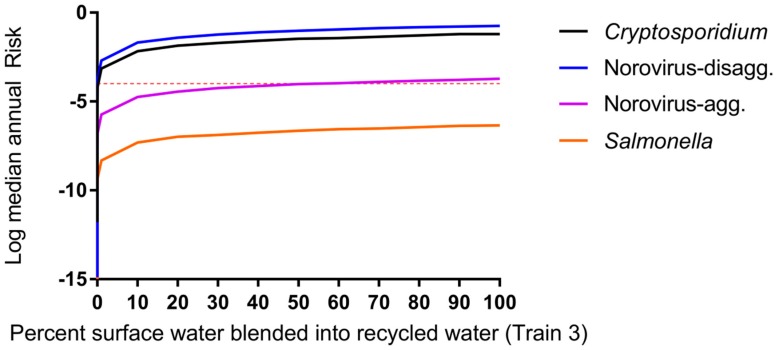
Impact of blending DPR water with 50% effluent-impacted surface water for treatment Train 3, case c, *F_dilution_* = 0.5, 0 < *F_blend_* < 1. Microbial risk is driven by surface water quality (not DPR water) for all scenarios (scenarios for train 2, 4, and 5 produced highly similar results and are shown in Supplemental Figure S1). Even blending 1% of impacted surface water increases consumer risk.

The impacts of blending 50% secondary wastewater effluent-impacted surface water (de facto reuse) with DPR water having underdone advanced treatment prior to drinking water treatment are shown in Figure 5 and Supplemental Figure S1. Blending with surface water at this point in the treatment process is currently used at the Big Spring, TX water reclamation plant [6]. Changes in water quality were greatest up to approximately 10% blending of de facto water with DPR water and demonstrate that small additions of de facto water (as low as 1%) have significant impacts on computed DPR risks.

## 4. Discussion

The idea of using wastewater to augment drinking water supplies has historically faced opposition from the general public, and blending advanced treated wastewater with surface water supplies is one way to demonstrate that the supply is not dominated with water of wastewater origin [67]. However, surface water supplies may already be impacted with wastewater effluent from upstream communities. Wastewater effluents discharged into environmental waters are not required to be subjected to advanced treatment such as reverse osmosis and various membrane-based technologies (33 UCS § 1342, 2002), so impacted rivers and surface waters can potentially contain higher concentrations of pathogens than advanced-treated waters. Under the conditions investigated, unintentional reuse (scenario (1)) posed risks of infection greater than the 10^−4^ benchmark for norovirus and *Cryptosporidium*, and blending this surface water with advanced-treated water resulted in similar risks, as the risks were dominated by the surface water. This analysis does not apply to communities that have access to relatively pathogen-free water sources through protected watersheds or deep aquifer groundwater, but is relevant for communities using surface water downstream of wastewater discharges. As changes in climate patterns make the supply of surface water less reliable and urban populations grow, the quality of the surface water used for blending will become an even greater concern. Strategies for mitigating risk could include monitoring the quality of the surface water used for blending. Direct potable reuse without surface water blending (or blending with high quality surface water) appears to present a lower risk of infection to the consumer by the target organisms modeled than de facto reuse. Many decisions regarding treatment units, cost, protection of public health and feasibility need to be made as the industry moves towards water reuse as a critical component of the urban water portfolio. 

This analysis provides a framework to compare different treatment scenarios by health risk as a tool for decision-making in the design of reuse projects. For a specific location, this analysis can provide a starting point for additional risk modeling using location specific pathogen concentrations. For each treatment train, the most representative removal and inactivation for the target pathogens was used. However, this analysis highlights the importance of additional information for pathogen inactivation under full-scale conditions, especially for viruses in NF and and RO, and protozoan and virus removals via UV, chlorine, and ozone. Because removal by disinfection processes is a function of the dose, better approaches for accounting for disinfectant dose are needed to allow full-scale removal data (which accounts for process variability) collected under one target dose to be extrapolated to other doses. These data gaps are especially notable as these processes were identified as highly influential on DPR train performance in a process-by-process assessment of health risks and ultrafiltration LRVs had to be used as surrogates for NF. In some cases, surrogates were used (such as MS2 or *E. coli*); while this practice is appropriate for QMRA, additional research is needed to quantify the removal/inactivation of actual pathogens under full-scale conditions. In particular, the aggregation state of norovirus can have a large impact on risk estimates [60]. Additionally, the removal and inactivation of norovirus by various unit processes may be different depending on its aggregation state as has been noted in some bench-scale studies indicating the importance of solution chemistry [68]. Aggregation state can have potential impacts on estimated process LRVs [69]. However, little information is available on the effect of aggregation for the various full-scale treatment processes of interest in the current Monte Carlo models. Therefore, the state of aggregation was considered for the dose response models but not the treatment scenarios (LRVs). The impact of the various full-scale treatment processes on potential aggregation of norovirus and resulting risks is a research gap that remains to be addressed. It is noted that log removal values for specific treatment facilities vary and the focus of this assessment was to examine comparative, rather than absolute risks. 

Limited information is available for actual blending ratios likely to be used, and the specific location of blending within the treatment train. This QMRA indicates that source water quality is an extremely influential parameter when blending advanced-treated DPR water with wastewater-impacted source water. Limiting the percentage of surface water impacted with sewage effluent may not be feasible depending on local conditions. This analysis also assumed background “pristine” waters and that there were no additional pathogen loads, such as from stormwater or agricultural sources. However, these inputs are common and could be included in a more detailed consideration of pathogens in source waters, accounting for fate and transport. The desired blending ratio is dependent on the source water quality; however, the current analysis indicates that for a source water quality impacted by 50% secondary wastewater effluent, a blending ratio < 1% can still have large impacts on modelled risks. 

The potential impact of recovery efficiencies of the various analytical methods used to measure pathogen removals was not considered in this analysis, and all studies used as input parameters considered the measured organisms to be viable and infectious. Therefore, comparing measurements made via culture (*Salmonella*) with those made via qPCR (norovirus) can introduce some inaccuracies in the model. However, the dose response models used for each of these organisms used culture and qPCR, respectively, indicating harmonization between these parameters. Still, overestimation of norovirus viability and infectivity, and underestimation of *Salmonella* or *Cryptosporidium* risks associated with culture or fluorescence-based methods could have occurred. 

In the cases considered, de facto risks exceeded benchmarks while advanced-treated DPR water risks were below these values. It was assumed that the log removal values modeled were representative of removal achieved in full-scale facilities, and furthermore that distributions for log removals encompassed typical variations in operation, maintenance, and design for the various treatment processes and can be generalized. Furthermore, we assumed that no changes in water quality occurred during storage or distribution; this is a limitation as these stages are known to impact water quality. In a similar analysis conducted by Soller et al. [12], risks were analyzed for various DPR treatment trains using uniform distributions of input parameters. The authors similarly noted annual risks below 10^−4^ for varying treatment trains and that the use of DPR can provide public health benefits when blended with conventionally treated waters. Results from a QMRA by Lim et al. [15] of de facto reuse in Trinity River, Texas also indicated that de facto risks exceeded traditional benchmarks, with the caveat that disease burdens may be within allowable limits using World Health Organization Disability Adjusted Life Year (DALY) benchmarks.

Finally, a key limitation and area for further exploration is regarding the assumption of independence of treatment processes and lack of catastrophic treatment failures considered in the current model. The reliability of various treatment approaches have been modelled for individual components including MBR [70] as well as overall reliability for general de facto reuse [71], water and wastewater treatment [72,73,74], and distribution system main breaks [75]. This analysis provides a flexible framework that can be combined with information on likelihood of treatment lapses in order to expand upon the current work using a dynamic probabilistic framework.

## 5. Conclusions

Data for pathogen removal by full-scale facilities were not available for all processes; surrogate information was used in several cases (MS2 for norovirus or *E. coli* for *Salmonella*, for example). Particular data needs include a method for determining disinfectant dose-specific inactivation values that capture the variability of disinfection processes (chlorine, ozone, UV) at full-scale; norovirus log removal by granular media filtration and RO; *Salmonella* removal due to RO treatment; *Cryptosporidium* removal in membrane bioreactors; and NF for all pathogens. Improving these data inputs will help to refine risk estimates and therefore provide more robust information for DPR risk management.

Modelled de facto reuse scenarios exceeded risk benchmarks (10^−4^) while DPR treatment trains were several orders of magnitude below the risk benchmark. Modeled infection risks for blending de facto reuse water with DPR finished water were driven by contamination levels in the surface water sources and not by the DPR treatment trains. Risks for de facto reuse were driven by the source water quality; with a source water quality impacted by 50% secondary wastewater effluent, a blending ratio <1% can still have large impacts on modelled DPR risks.

Analysis assumed no catastrophic integrity failures in the advanced treatment systems but incorporated typical process variability. This framework for analyzing health impacts of DPR can be expanded to include treatment failures (e.g., via dynamic probabilistic methods).

## Figures and Tables

**Table 1:** Treatment train scenarios examined for de facto reuse and direct potable reuse (DPR). Wastewater is shortened to ww. The log removal values used for activated sludge and membrane bioreactor (MBR) processes include primary sedimentation.

Train Scenarios	Cases Considered ^a^	Type	Source Water	Treatment Train Processes							
(1)	b, c	De facto	Wastewater impacted surface water	Coagulation/ sedimentation						Media filtration	chlorine
(2)	a, c	DPR	Raw ww	Activated sludge			MF	RO	UV/H_2_O_2_		chlorine
(3)	a, c	DPR	Raw ww	Activated sludge	Ozone	BAC	MF	RO	UV/H_2_O_2_		chlorine
(4)	a, c	DPR	Raw ww	MBR				RO	UV/H_2_O_2_		chlorine
(5)	a, c	DPR	Raw ww	Activated sludge	Ozone	BAC	MF	NF	UV/H_2_O_2_	BAC	chlorine

^a^ See Section 2.5 and Equation (3). **Case a:** *When F_blend_* = 0, only DPR trains are considered and no blending with de facto reuse source water is considered but DPR effluent undergoes additional conventional drinking water treatment; **Case b:** *F_blend_* = 1, only de facto reuse is considered; **Case c:** For 0 < *F_blend_* < 1, DPR water is blended with de facto reuse source water prior to conventional drinking water treatment. For cases b and c, 0 < *F_dilution_* < 1 where 0 is pristine, pathogen-free source-water, and 1 is source water comprised of 100% conventionally-treated wastewater effluent.

**Table 2:** Monte Carlo simulation input parameters for pathogen concentrations.

Parameter	Symbol	Unit	Value	Distribution	Source
Norovirus	*C_ww,raw,No_*	gene copies per L	µ = 9.095, σ = 1.413 × 10^−3^	Lognormal ^a^	[19]
*Salmonella spp.*	*C_ww,raw,Sa_*	Number per L	µ = 7.171, σ = 2.985	Lognormal	[22,23]
*Cryptosporidium*	*C_ww,raw,Cr_*	Number per L	µ = 2.262, µ = 0.944	Lognormal	[24]

^a^ Lognormal parameters mean, standard deviation (µ, δ) calculated from population (normal) parameters ($\bar{x}$, s) using standard formulae as follows: µ = ln($\bar{x}$^2^/(s^2^ + $\bar{x\;}$^2^)^1/2^), δ = [ln(1+( s^2^/$\bar{x}\;$^2^))]^1/2^, where $\bar{x}$ is the sample mean and s^2^ is the sample standard deviation.

**Table 3:** Monte Carlo parameters for treatment process log removal values.

Process	*Crytosporidium*	Reference	Norovirus	Reference	*Salmonella*	Reference
**Conventional drinking water treatment**						
Coagulation + sedimentation + granular media filtration	N (2, 0.5) ^a^	[25]	N (2.1, 0.7)	[26]	N (1.77, 0.25)	[25]
Chlorine disinfection	N (0.41, 0.4)	[24]	N (1.68, 0.24)	[27]	N (2.57, 0.35)	[27]
**Conventional wastewater treatment ^b^**						
Sedimentation + Activated sludge	N (1.58, 1.3) ^c^	[28]	N (2.1, 0.78)	[29]	N (3.32, 0.76) ^c^	[28]
Filtration	-	-	N (0.5, 0.02)	[30]	-	-
**DPR advanced treatment processes**						
Microfiltration (MF)	N (4.6, 0.96)	[31]	N (0.6, 0.1)	[32]	N (5.96, 1.47)	[31]
Reverse osmosis (RO)	N (4.5, 0.73)	[33]	N (4.3, 0.34)	[34]	N (6, 0.6)	[35]
UV	N (2.2, 1.17)	[36]	N (4.96, 0.85)	[37]	N (3.82, 0.34)	[27]
Ozone	1.2	[38]	5.4	[39]	4.15	[39]
Biological activated carbon filtration (BAC)	U (0,0.85) ^d^	[12,40,41,42]	U (0, 1)	[12]	U (0.5, 2)	[12,21]
Membrane bioreactor (MBR)	N (4.3, 0.6)	[43]	N (5.10, 0.41)	[44]	N (6.26, 0.75)	[27]
Nanofiltration (NF) ^e^	N (5.52, 0.51)	[45]	N (4, 0.1)	[32]	N (4.8, 0.6)	[46]

^a^ N = Normal distribution with parameters mean, standard deviation (µ, σ); ^b^ Undisinfected effluent used for input to DPR trains and disinfected effluent discharged to environment in de facto scenario; ^c^ Includes filtration; ^d^ U = Uniform distribution with parameters minimum, maximum; ^e^ Ultrafiltration values used.

**Table 4:** Monte Carlo simulation input parameters for exposure and dose response.

Parameter	Symbol	Unit	Value	Distribution	Source
Intake rate	*V_ing,dw_*	L per day	µ = −0.630, σ = 0.989	Lognormal ^a^	[63]
Exposure frequency	*n_dw_*	times per year	365	Point	Assumption
*Cryptosporidium* dose response	*r*	Unitless	4.19 × 10^−3^	Point	[9]
Norovirus dose response- aggregated	*P*	Unitless	0.72	Point	[59]
	*µ*	Unitless	1106	Point	
Norovirus dose response-disaggregated	*P*	Unitless	0.72	Point	[59]
	*µ*	Unitless	1	Point	
*Salmonella* nontyphoid dose response	*α*	Unitless	0.3126	Point	[9]
	*β*	Unitless	2884	Point	

^a^ Lognormal parameters mean, standard deviation (µ, δ) calculated from population (normal) parameters ($\bar{x}$, s) using standard formulae as follows: µ = ln($\bar{x}$^2^/(s^2^+$\bar{x}$^2^)^1/2^), δ = [ln(1+(s^2^/$\bar{x}$^2^))]^1/2^, where $\bar{x}$ is the sample mean and s^2^ is the sample standard deviation.

**Table 5:** Sensitivity analysis with Spearman rank correlation coefficients for DPR treatment trains.

Parameter	*Cryptosporidium*				Norovirus-Disagg.				Norovirus-Agg.				*Salmonella*			
	2	3	4	5	2	3	4	5	2	3	4	5	2	3	4	5
Wastewater concentration (*C_ww,raw_*)	0.17	0.16	0.24	0.14	0.002	0.005	0.011	−0.001	−0.006	−0.006	−0.002	0.0001	0.092	NA ^a^	0.277	NA
Contact rate (*V_ing,dw_*)	−0.004	−0.005	0.003	−0.003	−0.0004	0.002	0.004	−0.003	−0.001	−0.001	−0.005	0.005	0.0004		0.005	
Chlorine disinfection	−0.17	−0.16	−0.24	−0.14	−0.19	−0.17	−0.22	−0.16	−0.19	−0.05	−0.22	−0.04	−0.03		−0.09	
Conventional ww treatment	−0.56	−0.52		−0.45	−0.62	−0.62		−0.55	−0.61	−0.14		−0.12	−0.06			
MF	−0.41	−0.39		−0.33	−0.08	−0.06		−0.07	−0.08	−0.02		−0.02	−0.09			
RO	−0.32	−0.28	−0.44		−0.26	−0.24	−0.32		−0.26	−0.07	−0.31		−0.04		−0.15	
UV	−0.50	−0.47	−0.71	−0.40	−0.68	−0.61	−0.81	−0.61	−0.67	−0.15	−0.81	−0.12	−0.03		−0.08	
BAC		−0.10		−0.08		−0.21		−0.20		−0.06		−0.05				
MBR			−0.36				−0.39				−0.38				−0.17	
NF				−0.18				−0.07				−0.02				

^a^ NA = not applicable, risks below calculation limits; ^b^ For norovirus only.

## Supplementary Materials

The following are available online at www.mdpi.com/1660-4601/14/6/635/s1, Figure S1: Impact of blending DPR water with 50% effluent-impacted surface water. In all treatment scenarios, microbial risk is driven by surface water quality (not DPR water). Even blending 1% of impacted surface water increases consumer risk.
