# Supplementary material for: Drivers of Microbial Risk for Direct Potable Reuse and de Facto Reuse Treatment Schemes: The Impacts of Source Water Quality and Blending
Source: Int J Environ Res Public Health. 2017 Jun 13;14(6):635. doi: 10.3390/ijerph14060635 (PMC5486321; doi:10.3390/ijerph14060635)
Supplement: Supplementary file 1 [file ijerph-14-00635-s001.pdf]

SUPPLEMENTAL MATERIALS

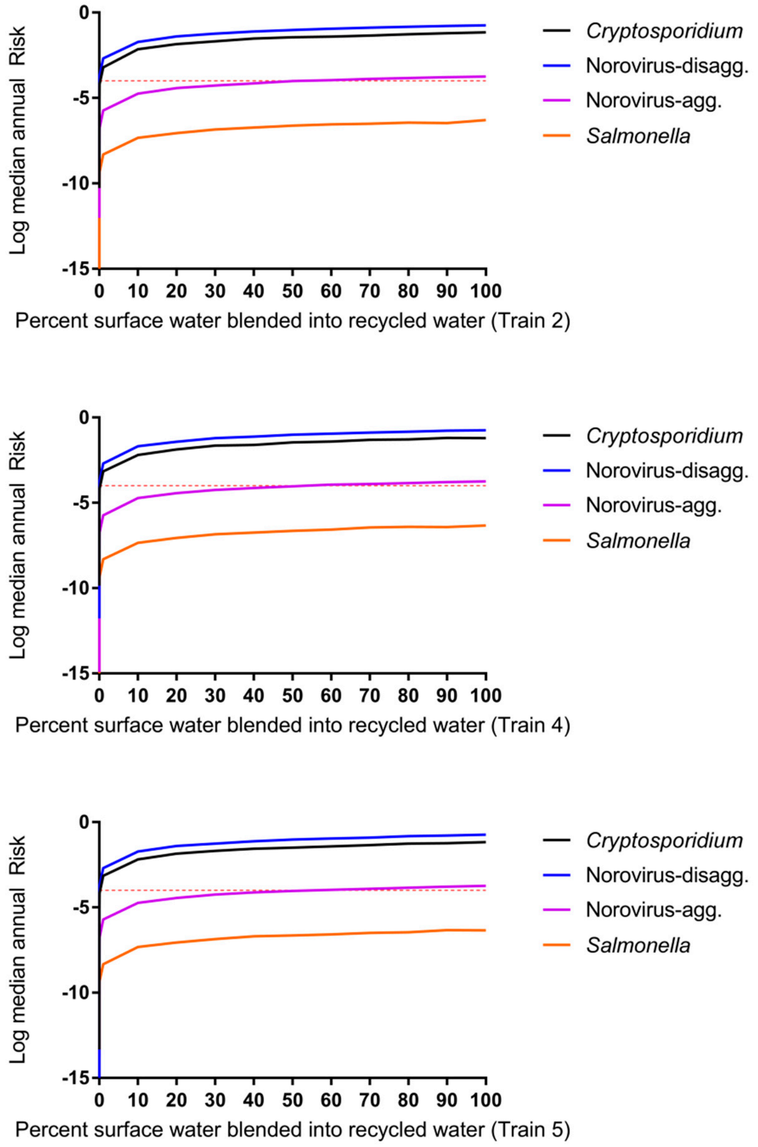

Fig. S1. Impact of blending DPR water with 50% effluent-impacted surface water. In all treatment scenarios, microbial risk is driven by surface water quality (not DPR water). Even blending 1% of impacted surface water increases consumer risk.
